# Complex and Messy Prebiotic Chemistry: Obstacles and Opportunities for an RNA World

**DOI:** 10.3390/life16020240

**Published:** 2026-02-02

**Authors:** Alberto Vázquez-Salazar

**Affiliations:** Departamento de Bioquímica, Centro de Investigación y de Estudios Avanzados del Instituto Politécnico Nacional (CINVESTAV-IPN), Av. IPN 2508, San Pedro Zacatenco, Gustavo A. Madero, Ciudad de México 07360, Mexico; alberto.vazquez@cinvestav.mx

**Keywords:** RNA world, prebiotic chemistry, origin of life, nucleotides, ribozymes, chemical evolution, messy chemistry, mineral catalysis, nonenzymatic replication, environmental selection

## Abstract

Traditional prebiotic chemistry experiments often isolated single reactions under clean, controlled conditions, yet early Earth was chemically diverse and physically dynamic. Such primordial complexity likely imposed obstacles, including side reactions, low yields, and unstable intermediates, but it also generated opportunities, including redundant routes, parallel pathways, and environmental filters that could bias mixtures toward subsets of persistent and chemically productive compounds. This review examines how heterogeneous prebiotic settings could generate RNA precursors, including nucleobases, ribose, and phosphate-containing species, through multiple concurrent pathways. Although side reactions can sequester carbon in inert tars and reduce yields of specific targets, networked chemistry can also enhance robustness when different routes converge on shared intermediates, or when apparent byproducts reenter productive cycles. Environmental factors such as ultraviolet irradiation, mineral surfaces, wet-dry cycling, and thermal gradients can act as constraints that enrich certain products by differential stability, reactivity, and compartmentalization. In this context, the RNA world hypothesis remains compelling, as RNA can store heritable sequence information and catalyze reactions through sequence dependent folding, thereby linking heredity and chemistry within a single polymer. At the same time, the emergence of functional sequence information and of control architectures that couple sequence to reproducible function remains a central open problem, and it sets clear limits on what chemistry alone can explain. Rather than dismissing messy mixtures as irrelevant noise, it is more accurate to treat them as the native context in which concentration mechanisms, environmental cycling, and selective persistence could enable the accumulation and survival of RNA related molecules.

## 1. Introduction

Studies on the origin of life have traditionally followed a reductionist path, often focusing on single reactions conducted under highly controlled conditions. Among the earliest and most influential examples are the experiments of Alexander Butlerow in the 19th century, who reported the spontaneous formation of sugars from formaldehyde in alkaline solution, now known as the formose reaction [1]. Nearly a century later, Joan Oró demonstrated that adenine, a key purine nucleobase, could be synthesized from hydrogen cyanide under relatively simple laboratory conditions [2]. These and related investigations, which gained prominence in the mid-20th century, helped establish the plausibility of abiotic synthesis of biologically relevant molecules and laid the foundation for modern prebiotic chemistry (Table 1). However, such studies often overlooked the inherent complexity of Earth’s early environment.

**Figure 1:**
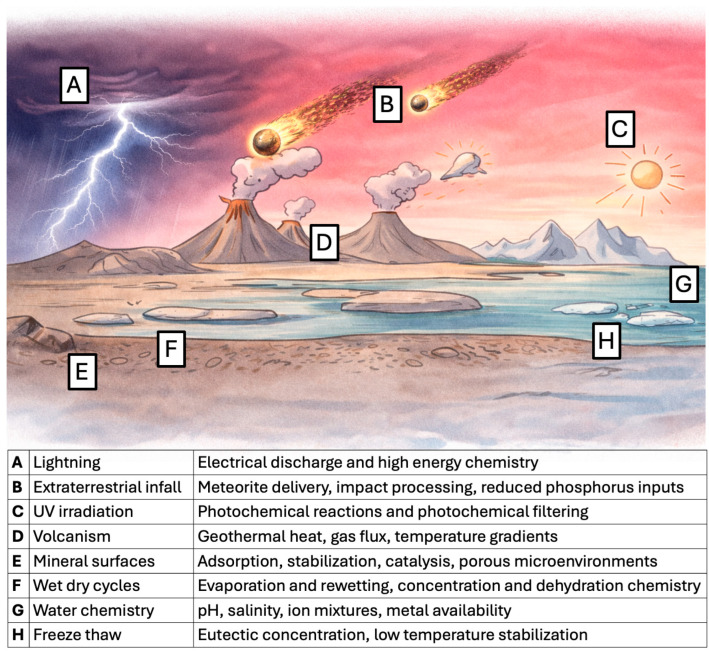
Environmental drivers that shape chemically complex prebiotic reaction networks relevant to RNA precursor synthesis, stability, and accumulation in heterogeneous early Earth settings. The panel illustrates major sources of energy and non-equilibrium processing that can concentrate, retain, stabilize, and filter products by differential persistence in complex mixtures. A, lightning and electrical discharge as high energy chemistry inputs, B, extraterrestrial delivery and impact processing supplying diverse organics and reactive phosphorus inputs, C, ultraviolet irradiation driving photochemistry and photochemical filtering, D, volcanism providing geothermal heat, gas flux, and temperature gradients, E, mineral surfaces enabling adsorption, stabilization, catalysis, and porous microenvironments, F, wet dry cycles promoting evaporation, rewetting, concentration, and dehydration chemistry, G, water chemistry as a boundary condition, including pH, salinity, ion mixtures, and metal availability, H, freeze thaw cycling enabling eutectic concentration and low temperature stabilization. The scene is conceptual and represents a mosaic of connected or intermittently connected niches rather than a single uniform environment.

Geological and astronomical evidence indicates that the Hadean Earth was chemically diverse and physically dynamic (Figure 1). Extraterrestrial infall delivered a broad array of organic compounds via comets and meteorites [3,4], and recent analyses of carbonaceous chondrites have revealed an unexpected variety of molecules, including all five canonical nucleobases of RNA and DNA [5]. At the same time, the Hadean Earth harbored a diverse mineralogical landscape, with estimates suggesting the presence of over 200 distinct mineral species [6]. These minerals were widespread and provided chemically active surfaces that could support adsorption, catalysis, and spatial colocalization of prebiotic molecules, processes that can increase local concentration and encounter frequency, and that can in some cases favor assembly into larger structures [7].

Environmental conditions on the early Earth were far from uniform. In the absence of an ozone layer, ultraviolet radiation penetrated to the surface; geothermal activity generated steep temperature gradients; and repeated wet-dry and freeze–thaw cycles acted on exposed environments. These shifting conditions likely generated diverse chemical niches, promoting the mixing, concentration, and transformation of organic mixtures across evolutionary time [8,9,10,11,12]. The resultant prebiotic chemistry was undoubtedly *complex*. Multiple reaction pathways would have operated simultaneously, generating a vast spectrum of products. Far from producing isolated and selective outcomes, such networks generated complex mixtures, a feature that has historically been viewed with skepticism.

As early as the 1980s, researchers such as Robert Shapiro questioned the prebiotic plausibility of key biochemical precursors arising in significant yield within uncontrolled chemical environments. In particular, the formose reaction, produces an assortment of sugars and polymeric byproducts, while generating only minimal amounts of ribose, the pentose sugar required for ribonucleotide formation [1,13]. Similarly, efforts to demonstrate the abiotic synthesis of cytosine and other nucleobases have often resulted in low yields and unstable products under plausible prebiotic conditions [14]. These limitations led some to argue that uncontrolled side reactions would divert carbon into chemically inert tars or “asphalts,” effectively inhibiting the emergence of biologically relevant molecules [14]. From this perspective, the inherent richness of prebiotic chemistry was not seen as a creative reservoir, but rather as a thermodynamic and kinetic obstacle, dispersing useful compounds into a background of reactive noise. However, recent advances in systems chemistry and experimental simulations have begun to challenge this view, highlighting mechanisms by which chemical complexity can be constrained, filtered, or even harnessed toward the formation of functional biomolecules [15,16,17].

Rather than viewing side reactions and competing pathways as liabilities, these models suggest that interconnected reaction networks can foster chemical robustness. Redundant pathways can converge on shared intermediates, while compounds that appear as byproducts in one step can participate in secondary reactions or reenter the network through recycling mechanisms. This notion draws upon earlier insights from metabolic control theory and is supported by recent prebiotic simulations that show how reaction networks, when coupled to explicit boundary conditions and degradation processes, can converge on constrained product subsets that persist under those conditions. Sutherland and colleagues, for instance, have shown that ultraviolet radiation, often considered destructive, can impose differential persistence by photolyzing some compounds more rapidly than others, thereby enriching key intermediates and functioning as a photochemical filter [18,19]. Similarly, mineral surfaces such as clays and metal oxides have been shown to promote the adsorption and protection of specific organic molecules, enhancing their local concentration and reactivity, while potentially sequestering inhibitors, thereby reshaping reaction fluxes in heterogeneous mixtures [6,7,20]. Consistent with the broader idea that compositional complexity can be productive rather than purely degradative, a long term Miller-type experiment that substituted distilled water with a seawater salt mixture supplemented with calcium phosphate and magnesium sulfate, and that maintained reactive gases through repeated regassing, yielded a broad suite of products in one apparatus, including amino acids, sugars including ribose, nucleic acid bases, nucleosides, lipids, oligopeptides, and detectable ATP [21]. Taken together, these findings suggest that under dynamic conditions including wet-dry cycles, thermal gradients, and fluctuating irradiation, reaction networks can yield constrained product sets and coupled reaction sequences, especially when concentration and degradation operate on different timescales [22,23,24,25]. In this context, terms such as selection and filtering refer to differential persistence imposed by the environment, and not to goal directed steering.

Within this evolving framework, the RNA world hypothesis continues to serve as a central, though actively debated, model for exploring the chemical origins of life [26,27]. Originally proposed in the late 20th century, the RNA world concept posits that before the emergence of DNA and proteins, early biochemical systems were based primarily on RNA molecules capable of both storing genetic information and catalyzing chemical reactions [28,29,30]. This idea gained traction following the discovery of catalytic RNA molecules, or ribozymes, which demonstrated that RNA is not limited to passive roles in genetic transmission but can actively participate in metabolism-like chemistry [31,32]. Here, sequence information is used in a minimal operational sense, the linear order of nucleotides that can be copied by base pairing and that determines folding and binding properties. The appeal of the RNA world lies in this dual functionality: RNA can act as a repository of heritable sequences and as a catalyst, facilitating reactions in the absence of protein enzymes [26,27]. In chemically diverse prebiotic environments, such versatility could have coupled molecular persistence to local chemical effects, a prerequisite for molecular evolution once copying and amplification became feasible [33].

Although this review concentrates on chemical plausibility, a complete origin of life scenario must also explain how functional sequence constraints and regulatory control could arise from initially stochastic polymers. The vast majority of random sequences are expected to be inactive or only weakly active, and the transition from heterogeneous polymer mixtures to populations enriched in replicative or catalytic sequences remains a key unresolved problem [34]. Accordingly, when this review uses terms such as information and organization, they refer to operational properties, heritable sequence patterns that can be copied and that can bias chemistry through folding and binding, rather than to semantic meaning or goal-oriented design.

Despite its conceptual strengths, the RNA world hypothesis faces several unresolved issues. Chief among these are the difficulties associated with prebiotic nucleotide synthesis, the polymerization of ribonucleotides into RNA chains, and the chemical instability of RNA under many environmental conditions [35,36,37]. Nonetheless, the model remains experimentally tractable and has inspired a wide array of investigations aimed at identifying plausible synthetic pathways, stabilizing conditions, and supporting environments [38,39,40]. More recently, the RNA world framework has been expanded by models that integrate proto-metabolic chemistry, suggesting that early RNA systems likely coexisted with simple metabolic cycles and interacted with peptides, lipids, and inorganic cofactors rather than operating in isolation [41,42,43,44]. These expanded views suggest that the RNA world may have been embedded within a chemically heterogeneous and dynamic setting, where mutually reinforcing interactions among different molecular classes facilitated the emergence of increasingly complex biochemical systems [27]. Rather than a stand-alone stage, the RNA world is increasingly treated as one phase within a broader continuum of molecular evolution, in which RNA-based functions coexisted with other chemistries and were later joined by DNA and protein-based systems.

This review examines how chemically complex and physically dynamic prebiotic environments could support the formation, persistence, and copying of RNA, and related genetic polymers, under conditions that were heterogeneous in space and time. It begins by summarizing current constraints on Hadean environments and the prebiotic atmosphere, and by clarifying how terms such as chemical complexity and plausible prebiotic are used when discussing multistep reaction networks that depend on local geochemical boundary conditions. It then surveys convergent routes to RNA precursors, including nucleobases, sugars relevant to ribonucleotide formation, and reactive phosphorus species, with emphasis on how minerals, irradiation, and concentration cycles can reshape product distributions in mixtures. The discussion then turns to RNA as a coupler of heredity and catalysis in chemically complex settings, covering plausible mechanisms for oligomer formation, environmental variables that modulate stability, and experimental progress on nonenzymatic copying, ribozyme catalysis, and RNA-peptide cooperation, including proto-chaperone-like contributions of short peptides, molecular midwives, and alternative routes to RNA-like heredity. The later sections broaden the focus to environmental obstacles and opportunities, examining how cycling conditions, gradients, interfaces, microdroplets, membraneless compartments, and ligation or recombination processes can hinder or enhance the persistence of functional sequences in crowded chemistries, and they conclude with experimentally testable questions framed in explicit environmental context.

## 2. Prebiotic Plausibility in Context, Environments, Atmospheres, and Chemical Complexity

A chemically diverse Hadean Earth is supported by geochemical, planetary, and experimental evidence, but it is increasingly difficult to treat the phrase prebiotic environment as a single, well-mixed global setting (Figure 1). Current origin of life models instead emphasize a mosaic of local regimes whose pH, redox state, ionic composition, temperature range, and water activity could differ strongly across space and time, and where each regime can enable only a subset of reaction classes while suppressing others [8,45,46]. In this manuscript, chemical complexity refers to the joint consequence of molecular diversity and reaction network connectivity, coupled to physical non-equilibrium processes that repeatedly concentrate, separate, and energize mixtures. Relevant drivers include wetting and drying, freezing and thawing, thermal gradients, irradiation cycles, and phase separation, processes that can shift both reaction rates and product persistence without implying that complex mixtures are intrinsically directed toward specific outcomes [16,47]. This framing highlights constrained fluxes and differential persistence in heterogeneous settings, rather than any assumption that complex mixtures are inherently self-organizing toward particular biological targets [47].

Comparable considerations apply to the prebiotic atmosphere, which set boundary conditions for feedstock availability and energy inputs. For much of the 20th century, strongly reducing atmospheric scenarios, often represented as mixtures dominated by CH_4_, NH_3_, H_2_O, with variable H_2_ and sometimes N_2_, were shaped by early proposals associated with the Oparin framework for a reducing primitive atmosphere, by thermodynamic and cosmochemical reasoning including equilibrium arguments developed by Urey, and by the early success of Miller-type electrical discharge experiments performed in reduced gas mixtures [48]. In contrast, geochemical constraints and atmospheric modeling increasingly support an anoxic atmosphere in which N_2_ was a major constituent and CO_2_ was abundant, while the abundances of reduced gases such as H_2_, CH_4_, and CO likely varied with mantle redox state, volcanic outgassing, atmospheric escape, and episodic events such as large impacts [48]. Because many canonical feedstocks, including hydrogen cyanide (HCN) and related nitriles, depend on high energy processing, it is useful to distinguish continuous atmospheric production from transient scenarios linked to impacts, volcanism, or other events [48]. Hypervelocity impact simulations provide one concrete mechanism by which short lived reducing conditions and high energy processing can generate HCN and yield downstream formation of nucleobases and simple amino acid products, establishing a bridge between atmospheric processing and surface inventories through deposition and runoff [49]. Subsequent aqueous chemistry also depends on local water composition, since plausible settings ranged from relatively dilute freshwaters to concentrated evaporite brines whose ion mixtures reflect atmosphere, water, rock interaction and evaporation history, with direct consequences for sugar stability, phosphate speciation, metal availability, and nucleic acid relevant condensation chemistry [50]. Many reaction steps discussed in this review are therefore best viewed as distributed across partially connected settings, including atmospheric synthesis and delivery, surface ponds and geothermal fields that supply cycling and irradiation, mineral rich interfaces that adsorb and concentrate solutes, and cold environments that generate concentrated eutectic phases, rather than being assigned to a single homogeneous soup [8,45,46,51].

The recurrent use of terms such as prebiotic and plausible prebiotic also requires explicit qualification. Recent analyses argue that plausibility is not a binary label, but a claim tied to stated assumptions, including the origin and concentration of feedstocks, the identity and accessibility of activating agents, the physical history of the setting, and continuity between steps, particularly the extent to which multistep sequences avoid hidden purification, externally supplied reagents, or mutually incompatible conditions [46]. This concern has motivated interstep compatibility tests that ask whether successive transformations can proceed under geochemically coherent constraints, treating environmental realism as an experimental variable rather than as an after-the-fact narrative [52]. These considerations clarify why discussion of messy prebiotic chemistry is inseparable from environmental modeling, because proposed pathways depend as much on where chemistry occurs and how mixtures are processed as on the intrinsic reactivity of isolated substrates [45,46,47].

## 3. Messy Pathways to RNA Precursors

**Figure 2:**
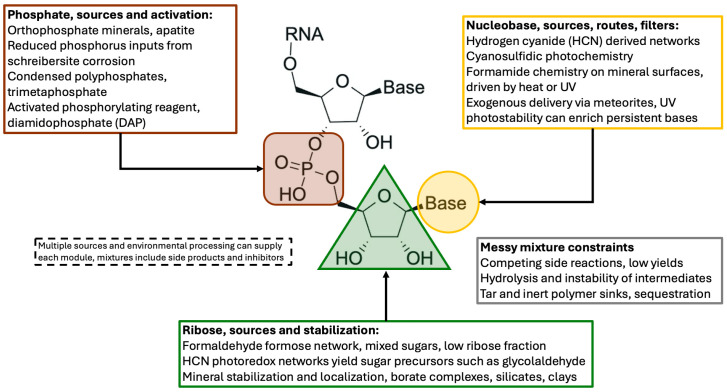
Modular assembly of RNA from heterogeneous prebiotic precursor pools under environmental filtering. The schematic decomposes RNA into nucleobases (yellow circle), ribose (green triangle), and phosphate (brown rounded square), and summarizes plausible sources and processing routes for each module in chemically complex settings. Nucleobases can arise from HCN derived networks, cyanosulfidic photochemistry, and formamide chemistry on mineral surfaces, with exogenous delivery and UV photostability contributing to selective persistence. Ribose and related sugars can derive from formose chemistry and HCN photoredox networks that supply sugar precursors, with borate and silicate or clay interfaces stabilizing and localizing sugars. Reactive phosphate species span orthophosphate minerals such as apatite and more reactive reduced or condensed phosphorus supplied by schreibersite corrosion, polyphosphates or trimetaphosphate, and diamidophosphate (DAP). Arrows indicate possible coupling and convergent supply of the three modules into nucleotide building blocks and short phosphodiester linked RNA segments. The gray box highlights mixture level constraints, competing side reactions, hydrolysis, and sequestration into inert polymers, emphasizing that assembly occurs in crowded mixtures where environmental processing shapes which intermediates persist.

Among the most persistent objections to the RNA world hypothesis is the considerable difficulty of synthesizing its monomeric components under plausible prebiotic conditions. Each ribonucleotide consists of three distinct molecular fragments: a nitrogenous base, a ribose sugar, and a phosphate group (Figure 2). The formation of these components, as well as their subsequent assembly into ribonucleotides, presents both thermodynamic and kinetic challenges, particularly in the chemically heterogeneous environments that likely characterized the Hadean Earth. These difficulties were already evident in early prebiotic studies, which demonstrated the synthesis of amino acids and simple carboxylic acids under simulated primordial conditions [53], but yielded limited success in generating nucleotides or their individual building blocks.

**Figure 3:**
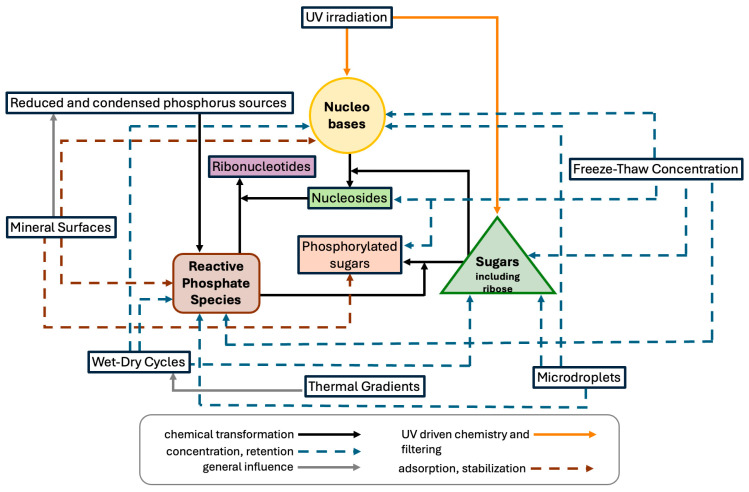
Schematic overview of how chemically complex prebiotic settings can support the formation and accumulation of RNA related monomers through coupled reaction networks and environmental processing. Central precursor pools, nucleobases (yellow circle), sugars including ribose (green triangle), and reactive phosphate species (brown rounded square), connect through chemical transformations to key intermediates, nucleosides and phosphorylated sugars, and to ribonucleotides as a monomer level outcome. Reduced and condensed phosphorus sources and mineral surfaces can expand access to reactive phosphate chemistry and reshape mixture composition through adsorption and stabilization, while wet-dry cycles, freeze-thaw concentration, thermal gradients, and microdroplets can concentrate and retain reactants and products, increasing encounter frequencies and enabling local enrichment across repeated processing. Ultraviolet irradiation can drive photochemistry and filtering by differential persistence within complex mixtures. Arrow styles indicate process classes, solid black arrows denote chemical transformation, blue dashed arrows denote concentration and retention, brown dashed arrows denote adsorption and stabilization, orange arrows denote UV driven chemistry and filtering, and gray arrows denote general influence.

Despite these limitations, a growing number of studies have shown that molecular complexity, far from being an impediment, may have facilitated the emergence of ribonucleotide precursors. In contrast to linear synthetic schemes, it is now understood that converging reaction networks can yield key intermediates through multiple routes, many of which operate simultaneously (Figure 2 and Figure 3). For instance, the work of Sutherland and colleagues has demonstrated that cyanosulfidic chemistry can give rise to precursors of sugars, amino acids, and nucleotides from common starting materials such as hydrogen cyanide, glycolaldehyde, and sulfite, when exposed to simple environmental inputs like ultraviolet irradiation and temperature fluctuations [44]. Similarly, formamide-based pathways have been shown to generate a variety of nucleobases, including purines and pyrimidines, under geochemically plausible conditions [54].

These reaction networks are shaped by environmental factors that influence product distribution, stability, and accumulation. Ultraviolet radiation, often considered a destructive force, has been shown to play a dual role: it drives key photochemical transformations and selectively degrades side products, thereby enriching more stable and functionally relevant molecules [10]. Mineral surfaces, such as borates and clays, can also act as selective agents by stabilizing ribose or enhancing the phosphorylation of organic substrates [55,56]. Under such conditions, parallel and intersecting reaction sequences may have generated localized chemical landscapes where biologically significant molecules accumulated despite the apparent disorder of the system. This revised view suggests that a complex environment need not be seen as a barrier to nucleotide formation, but rather as a chemically rich setting in which multiple synthetic opportunities could be explored and shaped by selective geochemical constraints.

### 3.1. Nucleobases

The synthesis of nucleobases under plausible prebiotic conditions has long attracted attention as both a productive and problematic aspect of origin of life research. These nitrogen rich heterocycles, adenine, guanine, cytosine, and uracil, are the components that allow RNA to function as an informational polymer, because their hydrogen bonding patterns and stacking interactions enable complementary base pairing and templating, which in turn allow heritable sequence patterns to be copied and maintained in populations of strands [57,58]. While abiotic formation is chemically feasible, many routes yield mixtures in which canonical nucleobases coexist with noncanonical heterocycles and chemically modified derivatives, a composition that complicates any simple narrative of direct incorporation into early genetic systems. At the same time, the existence of broad heterocycle inventories is not unexpected in chemically open reaction networks, and it shifts the central question from whether diversity existed to when and how selection began to act on that diversity. In this context, selection does not need to imply Darwinian evolution at the outset. Composition can be biased by differential persistence and differential availability imposed by the environment. Ultraviolet irradiation provides one such filter, since canonical nucleobases exhibit unusually efficient photophysical dissipation pathways that confer high photostability relative to many alternative heterocycles, a property that has been discussed as a potential constraint on surface exposed inventories under intense ultraviolet flux [18,59,60]. Aqueous reactivity can impose additional biases, because hydrolytic transformations such as deamination and related reactions can reshape heterocycle pools over time, altering which bases remain available for nucleoside and nucleotide formation under given solution conditions [60]. Partitioning also matters, because adsorption at mineral interfaces can concentrate heterocycles and nucleotides, while simultaneously imposing selectivity that depends on mineral identity, surface chemistry, exchangeable cations, and solution composition [61,62]. Retention within compartments can further bias availability, since phase separated droplets such as complex coacervates can concentrate and retain nucleic acids and related solutes, changing local concentrations, reaction kinetics, and effective persistence relative to bulk solution [63,64,65]. Once even inefficient template copying becomes possible, kinetic selection can add an additional layer of bias, because nonenzymatic primer extension tends to favor monomers that form near Watson–Crick geometries and allow continued extension, whereas mismatches often stall elongation and reduce the likelihood of producing full length copies, thereby skewing product distributions in mixed monomer pools [66,67,68]. Under this view, mixtures of nucleobases are not only an obstacle, they also provide the substrate on which repeated filtering, partitioning, copying, and retention can narrow the subset of bases that participate in persistent informational cycles, without assuming that any specific outcome was predetermined [60].

One of the earliest demonstrations of abiotic nucleobase formation was provided by Joan Oró in 1961, who showed that concentrated HCN in aqueous ammonia can yield adenine through oligomerization reactions involving the tetramer diaminomaleonitrile (DAMN) and other HCN-derived intermediates [2,69]. Although this result provided a striking example of molecular complexity arising from a simple one-carbon feedstock, it also illustrated the limitations of such systems, which tend to produce complex mixtures, including a variety of purine analogs and insoluble polymers.

At this point it is useful to separate two distinct claims that are sometimes conflated, the feasibility of producing purines or related intermediates in abiotic mixtures, and the spontaneous emergence of stepwise biosynthetic routes. Even when prebiotic reactions generate compounds that resemble constituents or intermediates of extant metabolic pathways, this does not imply that linear or cyclic biosynthetic sequences assembled and persisted as coupled fluxes, because continuity and elaboration of such chains depend on sustained catalysis and energetic coupling whose stability is difficult to account for without a hereditary apparatus [70].

Subsequent studies expanded the range of possible nucleobase precursors. Formamide (H_2_NCHO), a solvent with both amide and aldehyde functional groups, has emerged as a particularly versatile compound. When heated in the presence of mineral catalysts such as clays or phosphates, or exposed to ultraviolet radiation, formamide can yield purines and pyrimidines, including adenine, guanine, cytosine, and uracil, along with related heterocycles [69]. Saladino and colleagues have demonstrated that formamide-based chemistry is sensitive to environmental variables such as catalyst type, temperature, and irradiation source, producing relevant nucleobases under geochemically plausible conditions [54].

Photochemical processes likely played a significant role in shaping the composition of prebiotic mixtures. Ultraviolet light, although destructive under some circumstances, can also drive selective reactions by accessing specific electronically excited states and redox pathways [18]. Recent work by Sutherland has emphasized the importance of UV-driven photoredox chemistry in prebiotic systems, showing that such pathways can enrich product mixtures in key molecules, including sugars and lipid precursors, while selectively degrading competing byproducts, consistent with photochemical filtering in chemically crowded networks [18,19,44,71]. Analogous photochemical filters may have acted on nucleobase precursors. For instance, photolysis of cyanide in the presence of sulfite under UV irradiation has been shown to produce reduced carbon compounds, including sugar precursors and simple nitrogenous bases [19,44,71]. Laboratory simulations of ultraviolet irradiation on ice mixtures and early Earth analogs have also produced nucleobases or nucleobase analogs, supporting the idea that UV processing of interstellar and atmospheric ices could contribute to prebiotic inventories [72,73,74,75]. Geological constraints and radiative transfer modeling indicate that, in the absence of a protective ozone layer, the surface of early Earth received substantial ultraviolet flux at biologically active wavelengths, particularly in shallow waters and exposed surface environments, with attenuation dependent on water composition and optical depth [10,76,77]. These settings would have supported photochemical reactions alongside thermal and mineral mediated processes.

The possibility that multiple nucleobase synthesis routes operated concurrently is now widely accepted. Atmospheric chemistry, geothermal activity, and extraterrestrial delivery may all have contributed to a chemically diverse and spatially distributed pool of purines and pyrimidines. Meteorites such as the Murchison and Murray carbonaceous chondrites contain measurable amounts of adenine and guanine, as well as nucleobase analogs and degradation products [5,78]. Recent analyses using high-resolution mass spectrometry have revealed the presence of uracil and cytosine at part-per-billion levels in some carbonaceous meteorites, suggesting that even the less stable pyrimidines could arrive exogenously [5]. These findings support a redundant and overlapping model of nucleobase availability, where multiple sources contributed complementary inventories to prebiotic settings.

A notable example of productive chemical complexity is provided again by the Sutherland group, who reported a cyanosulfidic proto-metabolic network capable of yielding precursors to nucleobases, amino acids, and lipids from shared feedstocks such as HCN, hydrogen sulfide, and glycolaldehyde [44]. In this scheme, ultraviolet irradiation and staged reagent inputs act as boundary conditions that bias reaction fluxes, so that multiple classes of small, soluble intermediates can be generated from the same pool while some competing branches are curtailed [18,44]. Rather than implying that complex mixtures are intrinsically self-organizing toward any particular target, these results illustrate a simpler point, environmental constraints such as redox state, irradiation, and timing can shape product distributions, sometimes limiting the accumulation of insoluble polymers and maintaining access to reactive intermediates [44].

These observations challenge the assumption that side reactions necessarily preclude prebiotic synthesis. Overlapping pathways can coexist, and when different microenvironments favor different branches, the combined output can sustain intermittent availability of key intermediates even if each individual route is inefficient [16,44]. In such a scenario, endogenous synthesis, photochemistry, and extraterrestrial input represent parallel sources that can contribute to the same heterocycle inventory, increasing the likelihood that local concentrations of nucleobases occasionally reached levels compatible with subsequent nucleotide assembly and copying chemistry [5,44]. In this way, nucleobases may have arisen through multiple geochemical and astrophysical routes, each contributing distinct, and sometimes complementary, sources of the heterocycles required for RNA to function as an informational polymer.

### 3.2. Sugars (Ribose)

The prebiotic formation of ribose, the five-carbon sugar that forms the backbone of RNA, has long been considered a central challenge to the RNA world hypothesis. Unlike nucleobases, which can arise through relatively simple oligomerization reactions, sugars such as ribose are produced through more elaborate networks of chemical transformations. The most studied of these is the formose reaction, in which formaldehyde undergoes base-catalyzed polymerization to yield a complex mixture of sugars, including ribose [1]. In principle, this reaction can generate biologically relevant monosaccharides abiotically, but in practice, it produces a wide array of compounds including branched and linear sugars, sugar alcohols, and polymeric tars, with ribose appearing only as a minor and highly unstable component. In alkaline solution, ribose readily undergoes isomerization and degradation, particularly through the Lobry de Bruyn-Alberda van Ekenstein transformation, which interconverts aldoses and ketoses and facilitates further side reactions. These findings have historically fueled skepticism, most notably expressed by Shapiro, regarding the prebiotic relevance of the formose reaction as a plausible route to ribose under early Earth conditions [13].

Despite these difficulties, a number of advances have revealed how geochemical complexity may have acted not as a hindrance but as a filtering system that promoted the accumulation and stabilization of ribose. One of the most significant discoveries in this regard was the identification of borate minerals as selective stabilizers of ribose. Borate ions, such as those found in naturally occurring minerals like colemanite and ulexite, preferentially bind to cis-diols, a structural motif present in ribose, forming borate-ribose complexes that inhibit its degradation. Experiments incorporating borate into formose-type reactions have shown a marked increase in both the yield and stability of ribose, with borate acting as a protective agent that diverts ribose from side reactions and prevents its transformation into undesired products [55]. The geological plausibility of this mechanism is strengthened by observations of boron-containing minerals in early Earth analog environments. Notably, the Curiosity rover has detected boron in Martian clay deposits, providing indirect support for the presence of borate-rich aqueous environments on the early Earth as well [79].

Silicate minerals, particularly in the form of clays and aluminosilicates, may also have contributed to the formation and preservation of ribose and related sugars. In laboratory simulations, silicate surfaces have been shown to catalyze variations in the formose reaction and to form stable organo-silicate complexes with sugars, effectively immobilizing them on mineral surfaces [80]. The formation of sugar silicate adducts can reduce exposure of some sugars to bulk solution chemistry and can localize them within mineral microenvironments, effects that may alter degradation rates and downstream reactivity relative to fully aqueous mixtures [80]. Clays such as montmorillonite can adsorb simple sugars and, under appropriate conditions, can increase effective concentration at charged interfaces and constrain molecular orientations, changes that can shift the balance between condensation, rearrangement, and decomposition relative to reactions in bulk solution [61,81]. Under explicitly specified geochemical conditions, montmorillonite and related minerals can also promote oligomerization of activated nucleotides and other monomers, supporting a broader role for mineral interfaces in local concentration and spatial organization of reactions, with outcomes that can include greater persistence of some biopolymer forming processes in favorable settings. Mineral catalysis, however, is strongly contingent. Reactivity varies with mineral identity, prior activation and alteration, the identity and abundance of exchangeable cations, solution composition, and the history of hydration, drying, and other environmental cycles, so clays are best regarded as context-dependent experimental parameters rather than universal catalysts [7]. 

Environmental cycling, particularly wet-dry and freeze-thaw processes, adds an additional layer of differential concentration and differential persistence. In scenarios such as evaporating ponds or geothermal fields, soluble organics and inorganic ions can become progressively concentrated as water evaporates, increasing encounter frequencies among formaldehyde, borate, and silicate minerals. As borate precipitates, it can sequester ribose in crystalline or amorphous complexes, which can persist through dry intervals. Upon rehydration, for example, by rainfall or tidal influx, these complexes can release ribose back into solution, changing both concentration and chemical context relative to the preceding phase. Cycles of concentration, precipitation, and release therefore provide a plausible mechanism for episodic ribose enrichment in specific geochemical settings over time [82,83].

Rather than requiring isolated formation of ribose in high yield, this framework supports a scenario in which modest, spatially localized reservoirs of sugars arise intermittently, for example, as borate complexes or as surface associated species on silicates and clays. If such reservoirs occur within, or exchange material with, environments that also support nucleobase chemistry and phosphate activation, then sugar availability becomes a probabilistic and spatially structured variable rather than a single global bottleneck. A mosaic of geochemical niches could therefore generate heterogeneous distributions of sugars and related intermediates, and repeated cycling could bias which molecules persist and remain chemically available for subsequent reactions.

### 3.3. Phosphates

The incorporation of inorganic phosphate into organic molecules is widely recognized as a central bottleneck in prebiotic chemistry [84,85,86]. In RNA, phosphate groups link ribonucleosides into a polyanionic backbone and contribute to aqueous solubility and molecular recognition through electrostatic effect [84]. On early Earth, phosphorus was likely dominated by orthophosphate (PO_4_^3−^), present in minerals such as apatite (Ca_5_(PO_4_)_3_OH), with dissolved concentrations controlled by mineral solubility, pH, and ionic composition [85,87]. However, the phosphorylation of organic molecules in water presents thermodynamic and kinetic difficulties. In aqueous media, phosphate esters are prone to hydrolysis, and the condensation reactions required to form phosphodiester bonds are generally unfavorable without catalysis or activating agents. The problem is also geochemical, orthophosphate can be sequestered by precipitation with calcium minerals and by interactions with iron phases, although anoxic Fe(II) can enhance phosphate mineral solubility and increase dissolved phosphate in some settings [88].

Recent advances have highlighted alternative geochemical pathways that may have circumvented these limitations. A growing body of work indicates that early Earth phosphorus chemistry likely included reduced species that are more reactive than orthophosphate. Meteorite delivered phosphides, particularly schreibersite, corrode in water to yield mixtures containing phosphite and other reduced oxyanions, together with phosphate, pyrophosphate, and related condensed species, and these mixtures can participate in organic phosphorylation reactions [89,90,91]. Laboratory simulations demonstrate that schreibersite-derived phosphorus can phosphorylate a range of substrates, including nucleosides that are converted to nucleotides under mild aqueous conditions [92]. Geochemical evidence for reduced phosphorus species in early Archean marine environments further supports the idea that multiple oxidation states of phosphorus may have been available to prebiotic chemistry [93]. In addition to meteoritic inputs, lightning strikes can generate reduced phosphorus phases in fulgurites and contribute reduced phosphorus oxyanions to near surface environments, potentially widening the availability of reactive phosphorus on land [94].

In parallel, geological processes such as drying and geothermal heating have been proposed as mechanisms to overcome the energetic barrier of phosphorylation in aqueous environments. Heating orthophosphate-bearing minerals can generate linear and cyclic polyphosphates that contain phosphoanhydride bonds analogous to those used for energy coupling in modern metabolism [95]. Independent experimental work has also shown that heating apatite with other minerals can yield polyphosphates while partially reducing phosphate, supporting a role for high temperature mineral processing in widening the accessible phosphorus redox and condensation space [96]. Upon rehydration, polyphosphates can act as phosphorylating agents, and wetting and drying sequences offer a physically plausible means to couple condensation with subsequent transfer chemistry [86].

Prebiotic phosphorylation can also be promoted by small molecule phosphorylating reagents. Diamidophosphate (DAP) can be produced from trimetaphosphate and ammonia and phosphorylates nucleosides, amino acids, and lipid precursors under aqueous solution conditions or paste-like, low water activity conditions, and under similar regimes it can generate mixtures that include oligonucleotides, peptides, and liposomes within the same reaction system [97,98]. Recent experiments further show that wet-dry cycling and simple additives can substantially improve DAP-mediated nucleoside phosphorylation and influence regioselectivity, reinforcing the sensitivity of phosphorylation outcomes to hydration history and solution composition [99].

Evidence for phosphorylation in intermediate hydration regimes is expanding. Urea-based eutectic solvents and related low water activity mixtures can mobilize phosphate from otherwise sparingly soluble minerals such as hydroxyapatite and promote phosphorylation in a single reaction setting [100]. A physicochemical orthophosphate cycle has been described in which orthophosphate is stored in an activated intermediate and subsequently transferred to organic substrates under mild, water-depleted conditions, linking geochemical phosphate availability to repeated phosphate transfer without assuming biological energy carriers [101]. Related proposals treat imidazole phosphate as an ATP analog and histidyl peptides as organocatalysts for phosphate transfer, placing phosphate transfer reactions within wet-dry-driven cycling regimes rather than implying a linear pathway [102].

Phosphorylation and nucleotide formation intersect directly with advances in prebiotic nucleotide synthesis. A stepwise synthesis of activated pyrimidine ribonucleotides from small feedstocks demonstrated that phosphate can participate in multistep reaction sequences that avoid preformed ribose and free nucleobases [39]. Unified schemes have since been reported that generate both pyrimidine and purine ribonucleotides from shared precursors under conditions that combine chemical reactivity with physical processes such as irradiation and crystallization, where phase behavior and cycling can remove, concentrate, or stabilize intermediates within an evolving mixture, reducing reliance on repeated laboratory isolation [103]. Taken together, these results suggest that the phosphate problem is best framed as a network problem, multiple sources of reactive phosphorus and multiple environmental activation modes likely coexisted, allowing phosphorylation chemistry to proceed in localized settings and to intermittently supply phosphorylated intermediates relevant to RNA chemistry, without implying goal directed progression [85,86] (Figure 3).

## 4. RNA at the Interface of Copying and Catalysis in Chemically Complex Environments

**Figure 4:**
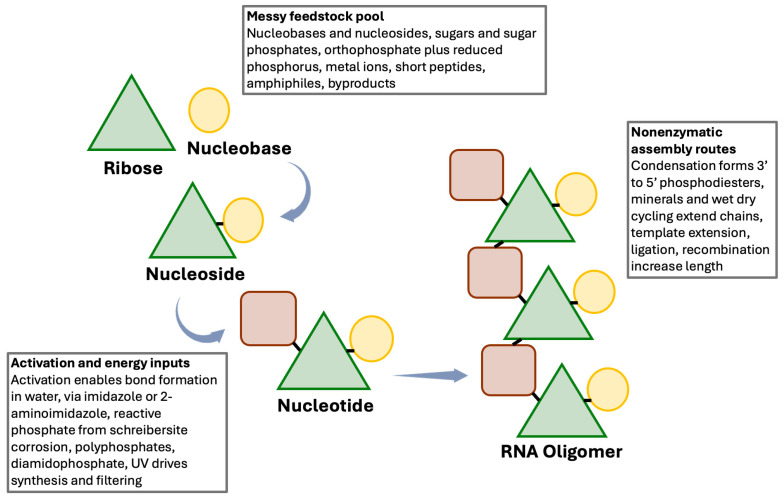
Schematic, modular route from a chemically messy prebiotic feedstock pool to RNA oligomers. Ribose (green triangles) and nucleobases (yellow circles), shown as separate components, can be coupled to form nucleosides, subsequent phosphate (brown rounded squares) addition yields nucleotides, and repeated activation plus condensation reactions can generate short RNA oligomers. The top box emphasizes that plausible starting inventories are heterogeneous, containing mixtures of nucleobases and nucleosides, sugars and sugar phosphates, orthophosphate plus reduced phosphorus species, metal ions, short peptides, amphiphiles, and diverse byproducts. The lower left box summarizes representative activation and energy inputs that can enable bond formation in water and in low water activity phases, including imidazole-derived activation chemistry, reactive phosphorus from schreibersite corrosion and condensed phosphates, diamidophosphate, and UV-driven synthesis plus photochemical filtering. The right box summarizes enzyme-free assembly processes that can increase polymer length, including wet-dry driven condensation on mineral surfaces, template-directed extension, and ligation, consistent with a view where accumulation depends on networks operating in mixtures and on environmental processing rather than on a single clean, linear pathway.

Assuming that the molecular components of RNA, nucleobases, ribose, and phosphate, were accessible on the early Earth through diverse geochemical pathways, the next major transition involved their assembly into functional polymers. These components were likely dispersed across multiple environments and seldom available in high purity, but could have been brought into contact through adsorption on mineral surfaces, environmental cycling, and phase separation processes that locally concentrate solutes and macromolecules [47,64,65] (Figure 4).

**Figure 5:**
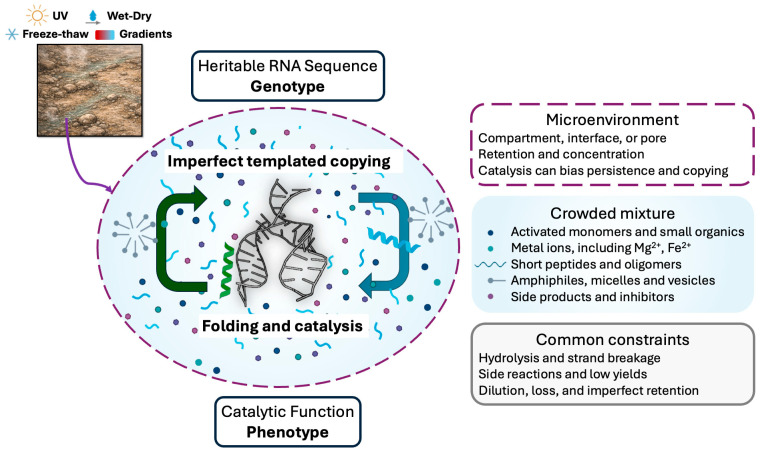
RNA genotype-phenotype coupling in chemically complex, environmentally-forced settings. Environmental drivers shown at top left, UV irradiation, wet-dry cycling, freeze–thaw concentration, and thermal and spatial gradients, represent non-equilibrium processes that repeatedly energize, concentrate, and reshape mixtures across connected microenvironments such as pores, interfaces, or mineral rich surfaces, illustrated by the inset. Within a local compartment, a heritable RNA sequence can undergo imperfect templated copying while the same sequence folds into catalytically active structures that alter reaction fluxes and can bias local persistence and further copying. Curved arrows indicate the reciprocal coupling between copying and catalysis. The dashed purple boundary denotes a local microenvironment that enhances retention and concentration. The crowded mixture includes activated monomers and other small organics, divalent metal ions, short peptides and other oligomers, amphiphiles forming micelles or vesicles, and diverse side products or inhibitors, emphasizing that functional sequences must operate amid competing chemistry rather than in isolation. Both Mg^2+^ and Fe^2+^ are depicted because divalent cations are central to RNA folding and catalysis while also influencing backbone cleavage. Fe^2+^ can substitute for Mg^2+^ and enhance ribozyme catalysis under oxygen-free, anoxic conditions relevant to early Earth, whereas extant biology predominantly relies on Mg^2+^. Common constraints include hydrolysis and strand breakage, side reactions and low yields, and dilution or loss due to imperfect retention. The RNA molecule depicted corresponds to PDB code: 1HMH.

The emergence of RNA as a polymer capable of both heredity and catalysis is a central premise in many origin of life models. In this review, sequence information refers to the linear order of nucleotides, an order that can, in principle, be copied by base pairing and that also constrains folding, binding, and catalytic potential. RNA can therefore couple heritable variation to chemical function, because different sequences can generate different structures, and some structures can accelerate specific reactions through well-defined secondary and tertiary organization [27,31,32,104]. Under chemically heterogeneous conditions, templating interactions can support imperfect copying, while catalytic activities can alter local reaction fluxes and material retention, for example, by promoting ligation, activation, or compartment level persistence, which links the relative abundance of particular sequences to their local chemical effects within a dynamic mixture [38,47]. In this framing, molecular evolution can begin before genetically-encoded protein enzymes, because selection can operate on differential copying, differential survival, and differential retention of RNA sequences across repeated physicochemical cycles, even when replication is inefficient and error-prone, and even when catalytic enhancement is supplied by RNA itself or by non-encoded partners such as ions, minerals, or short peptides (Figure 5).

This section examines how RNA relevant chemistry can be distributed across chemically complex environments and still generate polymer populations in which some sequences can, in principle, support both heredity and catalysis. It first discusses routes to assembling RNA oligomers from activated monomers, with emphasis on template-directed extension chemistries and on mineral assisted oligomerization on layered silicates and related interfaces [7,38,67,68,105,106,107,108,109,110]. It then evaluates physicochemical constraints on RNA persistence in water, focusing on how pH and temperature modulate backbone cleavage, and on how realistic ion mixtures, especially Mg^2+^ and Fe^2+^, jointly influence folding, catalysis, and degradation rates [111,112,113,114]. The discussion then turns to how catalytic activity and imperfect copying can be expressed and maintained in heterogeneous mixtures, including the contribution of small molecule or metal cofactors and the effects of microcompartments that concentrate polymers while remaining permeable to substrates, as in coacervates and related phase-separated media [47,63,64,65,115]. It also extends the analysis to non-encoded partners such as short peptides, which can modulate duplex stability, phase behavior, and reaction kinetics, and in some systems alter ribozyme performance through reversible association or condensate formation [116,117,118]. Finally, it evaluates proposals in which molecular midwives, reversible linkages, supramolecular assemblies, and alternative nucleobases or backbones reduce kinetic trapping and pairing bottlenecks, outlining intermediate regimes that may relax constraints on copying and strand retention without assuming canonical RNA as the only starting point [119,120,121,122].

### 4.1. Synthesis of RNA Polymers

Even if ribonucleotides were present, polymerization into RNA chains is difficult in the absence of enzymatic machinery. One strategy relies on chemically activated nucleotides, in which activation improves the leaving group properties of phosphate and enables phosphodiester formation under conditions where inactivated monomers are largely unreactive (Figure 4) [67,68,105]. Template-directed primer extension with activated monomers has been demonstrated in several experimental systems, and related condensation chemistries, including carbodiimide-mediated activation, can promote oligomer formation under mild aqueous conditions [106,107]. More recent work has shown that photochemical reaction networks can, in principle, couple nucleotide production with oligomerization under ultraviolet irradiation in one-pot settings, albeit yielding short products in modest amounts [18,25]. These results do not resolve all difficulties, but they establish that abiotic routes can connect synthesis and polymerization within plausible environmental contexts.

Mineral surfaces are widely invoked as additional contributors to polymerization by concentrating reactants and imposing orientational constraints at interfaces. Layered silicates, including montmorillonite, can catalyze the oligomerization of activated ribonucleotides and have been reported to yield RNA chains exceeding 50 residues under specific laboratory conditions [38,110]. However, the catalytic performance of montmorillonite and other clays is highly contingent on mineral identity, exchangeable cations, surface preparation, solution chemistry, and cycling history, so clay effects are best treated as context-dependent rather than universal catalysts [7]. Where the geochemical context is favorable and explicitly specified, mineral assisted polymerization remains a plausible route to heterogeneous pools of RNA strands that vary in length and sequence, providing a substrate on which selection could later act.

### 4.2. Stability of RNA

A frequently cited limitation of the RNA world hypothesis is the chemical instability of RNA, particularly under alkaline conditions and at elevated temperatures [111,112]. Backbone cleavage is promoted by the ribose 2′-hydroxyl group, which can drive intramolecular transesterification of phosphodiester bonds, and kinetic studies show that RNA lifetime varies over orders of magnitude as a function of pH, temperature, and ionic composition [111,112]. This intrinsic lability is tightly coupled to function, the same structural features that enable folding and catalysis also impose constraints on persistence, so the relevant question for prebiotic settings is rarely whether RNA is stable in bulk water, but rather which coupled chemical and physical regimes maximize the persistence of functional sequences long enough for ligation, copying, and retention to occur [113,121].

Ionic composition is central to this problem and is part of the messiness of any natural water source. Magnesium is particularly consequential because Mg^2+^ is a major contributor to RNA tertiary folding and to many catalytic mechanisms, through direct coordination to phosphate groups and through diffuse electrostatic screening that stabilizes compact structures [113]. At the same time, Mg^2+^ can accelerate strand scission by catalyzing in line attack of the 2’-oxygen on the adjacent phosphate, which generates a tension between activity-enabling and persistence-limiting roles for the same ion [113,123]. Recent experimental and theoretical work has formalized this tradeoff by showing that structured RNAs can exhibit a local maximum in chemical lifetime at intermediate Mg^2+^ concentrations, where folding mediated protection partially offsets Mg^2+^ promoted cleavage [123]. In addition, Mg^2+^ speciation cannot be treated as a minor detail, magnesium binds strongly to polyphosphate rich molecules, including ATP and related nucleotide species, so the fraction of free Mg^2+^ depends on the total phosphate inventory, ionic strength, and competing ligands present in solution [124]. This reinforces that assessments of RNA persistence and function require explicit ion mixtures rather than idealized single salt buffers [113,124].

Other divalent metals that were plausibly available in anoxic environments expand this landscape. Iron in the Fe^2+^ state can substitute for Mg^2+^ in promoting RNA folding and can enhance the rates of ribozyme catalysis under oxygen-free conditions, indicating that early RNA chemistry could have operated in a broader and more metal diverse regime than modern aerobic biochemistry typically assumes [125]. Iron can also support redox-active RNA catalysis, including electron transfer, providing a concrete example of how alternative metal cofactors could increase catalytic scope without requiring protein enzymes [114]. Boron, typically present as borate in aqueous settings, is also relevant, although primarily through upstream effects on nucleotide availability rather than by universal stabilization of RNA polymers. Borate can guide key steps in prebiotic nucleotide chemistry, including ribose phosphorylation, and recent efforts to evaluate inter-step compatibility of multi-step RNA synthesis models explicitly incorporate borate-rich and phosphate-bearing conditions consistent with plausible Hadean environments [52]. However, systematic tests indicate that borate minerals do not generally act as broad-spectrum stabilizers of RNA backbones, so boron is best treated as a context-dependent modulator of precursor pools and solution chemistry rather than as a generic solution to RNA degradation [126,127].

Environmental heterogeneity provides additional stabilizing niches that can shift the balance from degradation toward persistence and assembly. Mildly acidic waters can reduce base-catalyzed cleavage relative to alkaline solutions, consistent with proposals that buffered acidic environments, including volcanic settings and carbon dioxide rich waters, could extend RNA persistence while still permitting productive prebiotic reactivity [12,37,111]. Low temperature regimes further expand plausible stability windows by slowing hydrolysis while concentrating solutes in interstitial brines within ice, a combination that increases effective concentrations without requiring complete drying [11,128,129]. Experiments show that ribozyme activity and nonenzymatic copying can proceed in ice matrices, and eutectic phases can facilitate ligation and related assembly steps, illustrating that freezing can function as a physical selector that couples concentration with kinetic slowing [11,128]. In addition, thermal gradients and other non-equilibrium settings can spatially separate zones of synthesis or activation from zones of accumulation, allowing polymers to persist in local refugia while continuing to sample dynamic conditions that support chemistry elsewhere in the same environment [9,47]. Finally, persistence is not only a molecular property, it is also a systems property. Adsorption to minerals and partitioning into condensed phases can localize RNA and modulate exposure to hydrolytic conditions, but these effects remain strongly context-dependent and must be evaluated as functions of mineral identity, exchangeable cations, surface preparation, and cycling history, as stated before [7]. Likewise, membraneless compartments such as coacervates can retain RNAs and enhance effective lifetimes by concentrating reactants and sustaining catalysis in crowded microenvironments, even when absolute chemical stability is limited in bulk solution [63,64,65]. Taken together, these results support a view of RNA stability as an emergent outcome of coupled variables, pH, temperature, water activity, ion mixtures including Mg^2+^ and Fe^2+^, and partitioning processes that define the time windows in which RNA can accumulate, react, and be copied in chemically complex settings [47,113].

### 4.3. Catalysis and Replication by RNA

The RNA world hypothesis becomes chemically consequential when RNA populations begin to display heritable catalytic effects. Many ribozymes require tens of nucleotides, but catalytic activity has also been reported in much shorter sequences under defined conditions, suggesting that rudimentary function may not require highly elaborate polymers [130,131]. In a prebiotic pool generated by abiotic polymerization, most sequences would be inactive, but rare folds could catalyze ligation, cleavage, or activation reactions that increase persistence or propagation, initiating selection.

Cofactors can broaden the catalytic repertoire of RNA. Contemporary biology retains many RNA ligand interactions, exemplified by riboswitches, demonstrating that RNA can bind small molecules and metal ions with high specificity [132]. In vitro selection experiments have produced ribozymes that employ small molecule cofactors, including flavins, to catalyze redox chemistry, supporting the view that RNA can extend chemical capabilities by recruiting environmental components [133,134,135]. This possibility is relevant to prebiotic settings where organic mixtures, metal ions, and mineral surfaces would have been abundant and spatially heterogeneous, providing a reservoir of auxiliary chemistry that could be harnessed by ligand binding RNAs.

Replication places a stringent constraint on any RNA world scenario. Nonenzymatic template-directed copying has been demonstrated with activated nucleotides, but reaction rates and fidelities depend strongly on activation chemistry and solution composition. Nucleotides activated with 2-aminoimidazole show improved reactivity and copying performance compared with earlier activating groups [68]. Additional components can reduce tensions between copying chemistry and compartment integrity, for example, citrate and related chelators can moderate Mg^2+^ reactivity and facilitate template copying in the presence of fatty acid membranes [42,136]. These results indicate that copying performance is a property of mixtures and physicochemical regimes, and that added chemical diversity can sometimes reconcile incompatibilities that appear in simplified systems. In parallel, in vitro evolution has produced RNA replicases capable of extending templates and, in recent work, sustaining adaptive evolution in RNA-only systems, demonstrating that RNA can couple inheritance with catalysis under defined conditions [137,138,139].

If replication and amplification occur, the composition of an RNA population no longer reflects only formation and degradation chemistry, it also reflects differential copying, retention, and resource use. Under such conditions, heredity and selection can reshape molecular inventories, and sequence dependent catalytic activities can feed back on local chemistry by altering reaction fluxes, substrate availability, or compartment level persistence. In this view, RNA is not only a product of prebiotic complexity, it is also one of the components through which chemical diversity can become linked to differential persistence in replicating systems.

### 4.4. Proto-Chaperones, Peptide Contributions to RNA Synthesis, Stabilization, and Replication

Any discussion of an RNA world in chemically complex environments benefits from treating peptides as an expected component of prebiotic mixtures, rather than as late additions. Several experimentally grounded routes generate short peptides under conditions compatible with fluctuating aqueous settings, including chemoselective ligation chemistry in water and related pathways that can yield peptide products without enzymatic activation [140,141]. In such settings, peptides would not be single, purified species, but mixtures spanning varied lengths and compositions, and their functional effects should be evaluated as properties of ensembles rather than as properties of an idealized sequence [142]. This compositional diversity matters because short peptides can interact with RNA through multiple physical mechanisms, including electrostatics, stacking, and metal ion coordination, and these interactions can modulate folding, duplex stability, strand exchange, and the local physicochemical context in which copying and catalysis occur (Figure 5).

A minimal and experimentally tractable proto-chaperone effect is duplex binding and stabilization. Synthetic cationic oligopeptides bind RNA duplexes, and systematic variation in side chain length and charge distribution changes the strength of RNA association and the stability of the duplex state [143]. These results support a basic point that is often implicit in RNA world discussions but rarely made explicit, polycationic peptides can partially substitute for inorganic counterions by screening phosphate repulsion, thereby shifting RNA conformational equilibria. In prebiotic mixtures, such screening does not require a single optimal sequence, it can arise from a distribution of short polycations whose collective effect is to bias RNA populations toward more compact or more persistent structures under specific ionic and hydration regimes.

At the same time, recent work indicates that RNA binding need not rely on cationic side chains alone. In vitro evolution of an RNA binding protein domain constrained to a limited amino acid alphabet lacking positively charged and aromatic residues yielded variants that still bind ribosomal RNA, with binding supported by K^+^-and Mg^2+^-mediated ion bridging between glutamate side chains and the RNA sugar phosphate backbone [144]. This finding is significant for prebiotic discussions because it expands the space of plausible peptide RNA interactions to include acidic or noncationic sequences in metal rich waters, and it frames some peptide-RNA contacts as emergent consequences of ion speciation and coordination chemistry rather than as simple charge complementarity [142,144]. In this broader view, proto-chaperone behavior can arise from mixtures that include both polycations and peptides with substantial carboxylate content, provided that local ion compositions support reversible bridging and do not drive irreversible aggregation.

Beyond generic electrostatic screening, experiments indicate that peptide-RNA interactions can be mutually stabilizing in ways that are relevant to messy chemistry. In model systems that couple RNA with plausibly prebiotic proto-peptides, peptide association stabilizes folded RNA structures, while RNA association can increase the persistence of proto-peptides against degradative processes, producing reciprocal stabilization within the same mixture [116]. This type of reciprocity does not require a strict division of labor between genetic and catalytic polymers, and it provides an experimentally grounded basis for scenarios in which RNA and peptides jointly reshape the stability landscape, thereby changing the available timescales for ligation, copying, and selection.

Peptides also influence RNA chemistry indirectly by promoting phase separation and condensed microenvironments. Coacervates and related membraneless condensates form readily when anionic RNA encounters cationic peptides or polyions, and such compartments can concentrate RNA and small reactants while altering diffusion, water activity, and ion partitioning. Importantly, recent work emphasizes that compartment function depends on parameters that are themselves part of prebiotic messiness, including polymer multivalency, charge density, and ionic strength. Moreover, more prebiotically plausible polyion multivalency can improve compartment function and ribozyme performance, relative to highly multivalent polymers that tend to overtrap or gelate the system [145]. In peptide-RNA coacervates, reducing peptide charge density can increase ribozyme activity, in part by changing RNA material properties and by partitioning magnesium into the condensed phase, illustrating that peptide composition can regulate both RNA mobility and the local availability of catalytically important ions [146]. Because early Earth settings likely imposed hydration and salinity cycling, it is also notable that dehydration-rehydration cycles can shift coacervate phase behavior and compartment level function, implying that the same environment that promotes condensation reactions can also tune the lifetime and permeability of peptide-RNA compartments [147]. These condensed phases have additionally been proposed as plausible environments in which peptides explore conformational space under crowding and partitioning constraints, which may change the distribution of accessible peptide states relative to dilute solution [148].

At the level of specific RNA reactions, peptide effects are best described as context-dependent modulation rather than uniform enhancement. Hydrophobic cationic peptides can modulate RNA polymerase ribozyme activity through accretion onto the ribozyme, with outcomes that depend on ionic strength and the balance between productive concentration and inhibitory overbinding [118]. In peptide-RNA condensates, ribozyme catalyzed recombination and oligonucleotide assembly can be enhanced, supporting a mechanism by which short RNA fragments could be reorganized and assembled within a crowded, polycation-rich phase rather than requiring processive polymerization in bulk solution [117]. At the same time, not all ribozymes respond strongly to peptides, and recent measurements report weak or limited effects of prebiotically plausible peptides on a self triphosphorylation ribozyme, reinforcing the need to avoid assuming that any peptide mixture will act as a universal helper [149]. A complementary caution emerges from studies of ribozyme activity in peptide-RNA coacervates, where catalytic activity can be inhibited relative to dilute buffer, while variations in peptide sequence modulate the degree of inhibition and the net reaction yield, indicating that condensates can either support or hinder catalysis depending on composition and material state [150].

Additional peptide-based scaffolds broaden the range of plausible proto-chaperone mechanisms. Amyloid-like assemblies interact with nucleotides and can show selective binding to codon sized RNA segments, suggesting that peptide aggregates may impose length scale selectivity on RNA pools and potentially bias which RNA fragments are retained or presented for further chemistry [151]. Likewise, peptide conjugates bearing polyaromatic hydrocarbons can increase catalytic RNA activity, consistent with the idea that chemically complex prebiotic mixtures could have contained both short peptides and hydrophobic aromatic compounds, produced by overlapping abiotic processes and delivered from multiple sources. In this context, plausible means co-occurrence within the same local setting, plus physicochemical opportunities for association, noncovalent complexation, or sporadic coupling that generate amphiphilic peptide aromatic components. Such components can partition to interfaces or condensed phases and thereby change RNA hydration, folding equilibria, or local concentrations of substrates and ions, with measurable consequences for catalytic performance [152]. More broadly, recent perspectives emphasize that peptides can contribute to early catalysis by stabilizing cofactors, tuning local solvation and dielectric environments, and providing metal binding microenvironments, without requiring the full modern amino acid alphabet or fully folded enzyme architectures [142]. This framing supports a continuum of peptide effects that includes direct RNA binding, indirect compartment formation, and cofactor centered catalysis in the same chemically heterogeneous settings.

Peptides can be treated as plausible proto-chaperones in an RNA world embedded in chemically complex environments, not because they necessarily increase every reaction rate, but because they can reshape the balance between folding, persistence, and copying in ways that depend on mixture composition, ion speciation, and environmental cycling. This reinforces a coevolutionary framing in which RNA and peptides interact across shared physicochemical constraints, including ion partitioning, phase behavior, and the stability of polymers in realistic water sources, and in which metal ions can mediate productive peptide-RNA contacts even for peptides that are not strongly cationic [142,144].

### 4.5. Molecular Midwives and Alternative Routes to RNA-like Heredity

A substantial body of work has emphasized that the major bottlenecks for abiotic genetic polymers are not restricted to monomer availability. They also include physical and kinetic constraints on strand pairing, extension, and retention in water, constraints that become particularly acute in dilute and chemically crowded mixtures. Template-directed synthesis requires persistent base pairing and stacking, yet growing oligomers can be diverted by strand cyclization, intramolecular folding, and product inhibition, all of which reduce the fraction of material that remains available for further extension. One influential response to these constraints is the proposal that early genetic polymers may have relied on transient helpers termed molecular midwives, as well as on reversible backbone linkages that reduce kinetic trapping and permit exchange during copying and assembly [119]. This framing aligns with demonstrations that intercalation can suppress cyclization and promote polymer growth by stabilizing extended, stacked conformations, a physical mechanism that does not require highly specific catalysts and that can operate in heterogeneous aqueous mixtures [153].

A closely related contribution is the argument that the earliest informational polymers may not have been canonical RNA, but rather a succession of primitive genetic polymers in which the backbone, the nucleobases, or both differed from modern nucleic acids, with later transitions toward RNA once more demanding components became accessible [120]. Within this perspective, nucleobase analogs that form strong complementary pairs and that undergo glycosylation more readily than canonical bases become relevant, because they reduce the number of coupled improbabilities that must be solved simultaneously. In particular, nucleosides and nucleotides based on melamine and barbituric acid have been shown to form spontaneously in water from ribose or ribose 5-phosphate, while also exhibiting complementary base pairing that supports supramolecular assembly [121]. Related aqueous reactions can yield high fractions of beta-ribofuranoside products for 2,4,6 triaminopyrimidine, and mixtures that include complementary heterocycles such as cyanuric acid can generate micrometer scale assemblies, indicating that covalent nucleoside formation and noncovalent pairing can be coupled within a single crude mixture, without invoking extensive purification [154]. These studies do not establish that any particular heterocycle set was uniquely relevant on early Earth, but they provide existence proofs that noncanonical nucleobases can ease constraints on nucleoside formation and pairing in water, and can generate nucleotide-like building blocks that already possess strong recognition properties.

Work on supramolecular polymers further supports the idea that ordering processes could precede fully developed phosphodiester genetics. Water-soluble assemblies of paired and stacked heterocycles can display polymer-like behavior and repetitive recognition motifs, offering a chemically conservative route to proto-informational systems in which noncovalent pairing and stacking contribute to linearity and persistence before efficient covalent polymerization becomes available [122]. This emphasis on physically grounded organization also complements the broader shift toward integrated reaction networks that tolerate side products and operate in one-pot sequences, rather than relying on linear routes that require repeated isolation of intermediates, a point highlighted in discussions of progress toward simplified nucleoside chemistry in aqueous mixtures [155].

At the same time, work outside this research program reinforces that early nucleobase inventories were likely broader than the canonical set. Alkaline hydrothermal simulations can yield diverse noncanonical nucleobases, expanding the space of plausible base analogs and strengthening the expectation that early mixtures contained competing, and potentially interfering, heterocycles alongside any canonical precursors [156]. However, alkaline hydrothermal environments, although attractive for redox chemistry and mineral driven gradients, face well known constraints as candidate settings for the emergence of RNA-like heredity. High pH accelerates RNA backbone cleavage through base-catalyzed transesterification, and elevated temperatures further shorten lifetimes, which together reduce the plausibility of sustained persistence for RNA polymers in strongly alkaline, warm waters [35,111,112]. In addition, many reaction steps invoked in surface-oriented nucleotide scenarios, including ultraviolet-driven photochemistry and wet-dry cycling that promotes episodic concentration and dehydration chemistry, are not naturally supported in deep submarine settings, even if hydrothermal microporosity can provide some concentration and catalytic advantages [83,157]. One coherent alternative is that hydrothermal systems contribute feedstocks and metal-rich fluids, while the most polymerization relevant steps occur in connected but distinct surface or near surface environments where cycles, irradiation, and variable water activity are available, an idea consistent with analyses comparing the environmental fates of nucleobases and emphasizing the importance of localized settings such as ponds and intermittently wetted surfaces [158]. Furthermore, recent analyses of purine chemistry in origin of life contexts emphasize that purine reactivity and lesion-type transformations expand heterocycle diversity and may connect to extant metabolic routes, reinforcing that early mixtures likely contained many interconverting purines rather than a single tidy pathway [159].

If heterocycle diversity and alternative backbones were plausible features of early mixtures, a central question is how and when selection began to enrich the canonical RNA components observed in extant biology. In prebiological contexts, selection can begin as differential persistence and differential participation in productive reaction fluxes, without requiring replication in the full Darwinian sense. Several nonexclusive filters have been proposed. One is photochemical selection, because surface environments likely experienced significant ultraviolet flux and the canonical nucleobases display unusually efficient nonradiative dissipation pathways that limit photodecomposition relative to many analogs, a property frequently discussed as a potential contributor to their enrichment under irradiation [60,160]. Another is network-level chemical selection, where multistep reaction sequences include intrinsic pruning steps, for example, selective precipitation, selective photoreduction, or selective degradation, that narrow product distributions and privilege intermediates that can enter subsequent transformations, a logic that has been used to explain how canonical ribonucleotide precursors can emerge from complex mixtures in integrated reaction schemes [39,103]. A third filter arises during copying itself. Experiments on nonenzymatic primer extension and related processes show that heterogeneous nucleotide pools do not necessarily remain heterogeneous after reaction, because chemical kinetics and duplex geometry can favor incorporation of certain monomers and disfavor others. In particular, nonenzymatic copying and extension reactions can enrich ribonucleotide content relative to arabino- and deoxy-analogues, supporting a model in which RNA can emerge from mixed nucleotide inventories through iterative cycles of extension and selection at the level of chemical reactivity and duplex compatibility, prior to the existence of protein enzymes [161,162].

Once any system achieves repeated copying with heritable variation, selection can shift from differential persistence of monomers and oligomers to differential propagation of sequences and ensembles. At that stage, monomer chemistry becomes indirectly selected through its effects on copying fidelity, folding stability, and catalytic performance. Canonical bases then become plausible attractors not because they are the only heterocycles that can form, but because they can jointly satisfy multiple constraints at once, predictable pairing geometry, compatibility with aqueous stability windows, and participation in catalytic folds, while competing bases that introduce frequent mismatches, disruptive tautomerism, or excessive photodamage would tend to reduce propagation under repeated copying regimes [60,120]. In this sense, the transition from pre-RNA candidates and supramolecular assemblies to canonical RNA-like heredity can be framed as a staged enrichment process, where early chemical and physical filters shape mixtures, and later copying-dependent selection consolidates those components that best support persistence, replication, and evolvability in chemically complex settings.

## 5. Messy Environments: Obstacles and Opportunities

The preceding sections emphasize a central point, prebiotic chemistry did not unfold in a single, well-mixed reactor, it occurred in physically structured settings far from equilibrium, where gradients, phase changes, and cyclic forcing continually reshaped concentrations and reaction options [47]. Within such settings, chemical complexity is ambivalent. It can dissipate scarce intermediates into side products and recalcitrant polymers, yet it can also impose constraints and sorting processes that bias product distributions, for example, through selective degradation, adsorption to mineral surfaces, or partitioning into condensed phases. Treating messiness as both obstacle and opportunity helps relate laboratory pathways to plausible environmental contexts, because it places the emphasis on reaction networks coupled to physical processing, rather than on isolated syntheses in homogeneous solution. In such coupled systems, the combination of cycling and compartmentalization can maintain access to reactive intermediates even when any single pathway is inefficient, because repeated concentration, spatial localization, and differential stability can favor continued participation of some compounds while others are removed from circulation. This framing does not imply goal-directed organization, it highlights that physical constraints can bias chemical ensembles and generate regimes in which differential persistence becomes consequential. A remaining challenge is to identify which combinations of reagents, cycles, and interfaces yield reproducible collective outcomes, such as sustained enrichment of particular intermediates, stable compartment formation, or self-reinforcing reaction loops, outcomes that are not apparent when individual reactions are studied in isolation and therefore require integrated, multivariable experimental designs.

### 5.1. Hindrances in Heterogeneous Reaction Networks

A chemically rich environment entails a proliferation of competing reactions. Reactive carbonyl compounds can be diverted into uncontrolled polymerization, amino compounds can intercept activated intermediates, and sugars and nucleobases can form multiple adducts that are not obviously on routes to informational polymers [13,14,15]. Such diversification can reduce yields of any single target compound and may immobilize carbon in complex macromolecular material that is difficult to recycle on experimental timescales, a concern long emphasized in critiques of prebiotic nucleotide chemistry [13,14,15]. These difficulties are not merely practical inconveniences. They reflect the underlying thermodynamic preference of dilute aqueous systems for hydrolysis and dispersal, together with the kinetic accessibility of side reactions once multiple functional groups coexist in the same mixture. In this sense, messiness generats a combinatorial burden, plausible intermediates must persist long enough and at sufficient local concentration to encounter compatible reaction partners, while avoiding rapid diversion into sinks.

### 5.2. Cycles, Gradients, and Interfaces as Natural Filters

Despite these limitations, dynamic environments provide mechanisms that repeatedly concentrate reactants, bias product distributions, and introduce selection by differential persistence. Hydration and dehydration cycles, particularly in terrestrial settings where evaporation and rewetting are frequent, can drive condensation chemistry during the dry phase and redistribute products during the wet phase, thereby coupling synthesis with recurrent mixing and dispersal [83]. Recent experiments have strengthened this physical logic by showing that heated wet-dry cycling can polymerize nucleotide monomers into long phosphodiester-linked chains, illustrating that cyclic forcing can substitute, in part, for continuous enzymatic catalysis [24]. Temperature cycling also offers opportunities. Freeze–thaw processes can concentrate solutes into eutectic brines, slow hydrolysis, and promote association reactions that are disfavored in dilute liquid water, including nonenzymatic nucleic acid assembly steps [11,128]. Irradiation cycles introduce a further layer of selection, ultraviolet light can be destructive, yet it can also drive photochemical routes and impose differential stability filters on mixtures, an effect that becomes relevant only in environments where illumination, shielding, and turnover vary in space and time [10,18]. The common feature across these scenarios is non-equilibrium forcing, the system is repeatedly displaced from chemical steady states, allowing reactions that are negligible under static conditions to become consequential over many cycles.

Spatial gradients provide complementary sorting mechanisms. In porous media subject to thermal gradients, convection and thermophoresis can concentrate dilute solutes and generate size- and length-dependent retention, generating microenvironments where polymerization and accumulation reinforce each other [9,47]. Mineral interfaces can contribute additional selectivity, both by adsorption and by catalytic organization of reactants. When parameters like mineral identity, activation state, exchangeable cations, solution composition are favorable and explicitly constrained, montmorillonite can catalyze polymerization of activated nucleotides into long oligomers, illustrating how surfaces can promote molecular ordering in localized settings while leaving bulk solution chemistry comparatively unproductive [38,110].

### 5.3. Microdroplets and Compartmentalization

Interfaces are not limited to rocks and sediments. Aerosols, sprays, and microdroplets generate transient compartments with high surface to volume ratios and rapid evaporative concentration. In laboratory simulations, aqueous microdroplets have been shown to promote the formation of sugar phosphates and, under specific conditions, uridine, reactions that are strongly disfavored in bulk water, suggesting that droplet environments can act as microreactors that couple dehydration, concentration, and interfacial organization [163]. Related droplet-based work has also demonstrated abiotic nucleoside synthesis through chemistry analogous to salvage pathways, supporting the broader idea that compartment scale physics can open reaction channels that appear inaccessible in dilute solution [164].

Membraneless phase-separated compartments offer a more persistent route to localization. Complex coacervates can enrich nucleic acids and other polyelectrolytes by partitioning them into dense phases, thereby increasing effective concentrations and altering reaction kinetics. Experiments have shown that ribozyme reactions remain viable, and can be enhanced, within compartmentalized crowded phases, consistent with the view that confinement and partitioning can transform weak chemistry into functionally relevant rates [63,64]. Moreover, RNA-peptide coacervates can exhibit feedback between RNA catalysis and droplet material properties, hinting at pathways by which catalytic function and compartment behavior could become coupled during early evolution [165]. Reviews that integrate physical principles with extant biological analogies further support a role for RNA containing membraneless compartments in origin of life chemistry, particularly as selective concentrators that remain permeable to small substrates while retaining larger polymers [47,65].

Recent work has begun to move from the claim that coacervates are compatible with RNA chemistry to a more mechanistic picture in which condensate composition and material state modulate reaction directionality and molecular exchange. In peptide-RNA condensates, charge-mediated phase separation can shift ribozyme-catalyzed equilibria toward ligation and promote oligonucleotide assembly and recombination, an effect that is consistent with increased local RNA concentration and altered microenvironments within the dense phase [117]. Complementary observations indicate that active ribozyme sequences can in turn modify droplet behaviors, including changes in wetting, growth dynamics, and RNA exchange between droplets, a set of effects that provides a physically grounded route to sequence dependent differential persistence at the compartment level [64,65]. Together with earlier demonstrations that coacervates can selectively retain longer RNAs while allowing exchange of smaller oligonucleotides, these advances strengthen the case that membraneless compartments can act not only as passive concentrators but also as substrates for feedback between function and compartment properties, a theme that aligns naturally with the broader emphasis on chemical complexity in prebiotic settings [63,64,65,165].

### 5.4. Ligation and Recombination as Routes to Complexity

A persistent challenge for RNA-centered scenarios is the emergence of sufficiently long sequences to support robust function, especially if direct polymerization yields are modest. One solution is modular assembly, short oligomers can be joined by ligation and recombination, allowing longer functional RNAs to arise from fragment pools. Template-directed, enzyme-free ligation systems demonstrate that copying and extension can proceed by joining activated short oligomers, rather than requiring fully processive polymerization from monomers [166,167]. In parallel, group I intron systems have provided concrete examples of covalent self-assembly and recombination. The Azoarcus ribozyme can be reconstituted from inactive oligonucleotide fragments through recombination chemistry, illustrating how fragmentation and rejoining can generate catalytic structures from heterogeneous pools [168,169]. Dehydration and rehydration cycling can further favor such assembly by repeatedly concentrating reactants and shifting equilibria toward joined products, an effect demonstrated in studies of ribozyme self-assembly under cycling regimes [170]. Beyond single molecules, experimental systems have shown spontaneous formation of cooperative RNA networks, where multiple replicators or fragments participate in mutually reinforcing cycles, suggesting that early selection could act on ensembles and pathways, not only on isolated sequences [171].

Taken together, these results support a conservative conclusion. Messy environments impose real chemical costs through side reactions, dilution, and instability, yet the same environments supply physical processes that can concentrate, filter, and compartmentalize mixtures, thereby generating pathways toward functional molecular organization. The origin of life problem is therefore not reduced to identifying a single optimal reaction sequence, it requires identifying the classes of environments and physical regimes that repeatedly bias complex mixtures toward persistence, joining, and replication of informational polymers [47].

## 6. Conclusions and Outlook

Any scenario for the origin of life must confront a basic feature of Hadean Earth, chemical inventories were heterogeneous mixtures shaped by cycling, gradients, interfaces, and compartments [16,47]. Chemical complexity can reduce yields through side reactions and competing sinks, and it can sequester carbon in poorly reactive products. At the same time, the same complexity can increase robustness when different routes converge on shared intermediates, and when environmental constraints, such as irradiation, phase changes, and mineral partitioning, impose differential persistence on mixtures [6,9,11,15,16,17,18,64,65,128]. In this context, selection can be defined operationally as differential persistence and differential enrichment imposed by physicochemical constraints in the absence of genetic inheritance. For example, molecules and assemblies that better resist hydrolysis or photolysis, that partition into minerals or condensed phases, or that remain concentrated within cycling microenvironments can accumulate relative to less stable or less retained alternatives. These filters act through differential stability, concentration, and reactivity, thereby biasing mixture composition and establishing initial conditions for later stages in which copying, compartment retention, and variation can sustain Darwinian dynamics.

Within this framework, the RNA world remains a productive, experimentally tractable hypothesis as RNA can couple heritable sequence patterns with catalytic activity in a single polymer. This dual role provides a plausible route to link copying and function without presupposing genetically encoded proteins. Early proposals that genetic polymers preceded encoded proteins, and that nucleic acids could combine informational and functional roles, were articulated before the term RNA world was coined [172,173,174]. Recent work on RNA-peptide coacervates and related membraneless compartments provides concrete mechanisms by which this coevolution could be physically realized, by concentrating RNA, short peptides, and cofactors while preserving catalytic activity [64,65,165]. However, the same framing also clarifies what remains unresolved. The discussion of RNA as an information carrier is meaningful only in an operational sense, information refers here to sequence dependent properties that can be copied by templating and that can bias folding, binding, and reaction outcomes. The emergence of functional sequence constraints and of control architectures that reliably couple sequence to reproducible function remains a central open problem, and it is not solved by chemistry alone. The RNA world hypothesis is therefore a plausible molecular substrate for early evolution while leaving open the mechanisms by which increasingly sophisticated regulation and coding systems arose.

Recent progress has nevertheless tightened the chemical plausibility space. Pathways to activated ribonucleotides and to shared precursor networks that connect nucleotides with other building blocks strengthen the case that RNA precursors could be supplied under realistic conditions [39,44,103]. Advances in nonenzymatic copying and activation chemistry, including improved activating groups and conditions compatible with compartment models, clarify how templated extension might proceed without enzymes [42,47,67,68,105,109,171,175,176]. Polymerization routes that exploit wet-dry cycling and mineral interfaces continue to refine constraints on where and when longer oligomers can accumulate, while also reinforcing a caution already emphasized in this review, mineral catalysis is context-dependent and must be evaluated as a parameterized geochemical effect rather than as a universal solution [7,38,110]. Laboratory evolution of RNA replicators provides an existence proof that RNA can, in principle, sustain heredity and adaptive change once sufficiently capable catalysts exist, even if current systems reflect intensive experimental optimization [137,138,139].

A realistic outlook also benefits from integrating RNA with peptides and cofactors. Multiple lines of work support the view that early evolution may have involved coevolution between RNA and short peptides, rather than a strict separation between an RNA stage and a later protein stage. Contemporary biology preserves extensive RNA ligand interactions, and experiments show that ribozymes can recruit small molecules and metals to expand catalytic range. Cofactor-mediated RNA catalysis deserves renewed attention, because modern biology offers multiple examples in which RNA binds small molecules and metals to extend functional range, and laboratory selections have shown that ribozymes can perform chemistry with additional substrates and cofactors [104,105,133,176]. In addition, peptide RNA condensates and related membraneless compartments provide physical mechanisms for concentrating and stabilizing functional assemblies, and for coupling catalytic activity to compartment properties, a minimal form of feedback relevant to early evolution [63,64,65]. The ribosome itself is consistent with this coevolutionary view, because the core chemistry of peptide bond formation is carried out by RNA, supporting the plausibility of translation related functions emerging in RNA rich contexts before modern protein enzymes dominated.

Looking forward, the most informative experiments are likely to be those that integrate chemistry with environmental context rather than testing reactions in isolation [47]. Ribozyme function and nonenzymatic copying should be challenged in chemically complex mixtures that include realistic competitors, minerals, fluctuating hydration, and variable ionic conditions, so that robustness can be quantified rather than assumed [9,83,93,109,175,176,177,178]. The same principle applies to cofactor-assisted catalysis, where candidate prebiotic cofactors should be tested as enabling components rather than treated as optional embellishments [115,133]. Equally important is the assembly problem, because short oligomers are easier to form than long ones, and routes based on ligation, recombination, and network level cooperation may provide more plausible pathways to functional length than processive polymerization alone [166,167,171]. Finally, coupling phosphorylation and activation chemistry to copying inside compartments, using environmentally plausible sources of chemical energy and cycling conditions, remains a key target because it connects geochemical energy flow to heredity, the minimal core of evolutionary dynamics [42,86,97,101,102].

Messy prebiotic chemistry should be treated as the realistic starting point for origin of life research, not as an experimental inconvenience [179]. The same heterogeneity that generates side reactions also generates concentration mechanisms, compartments, and persistence filters. The RNA world hypothesis, especially when viewed through models of RNA-peptide coevolution, remains valuable as it identifies a plausible molecular substrate in which copying and catalysis can coexist. At the same time, explicit recognition of the informational and control challenges clarifies what must still be explained, namely, how functional sequence constraints and reliable regulatory coupling emerged from initially heterogeneous polymers and fluctuating environments.

## Figures and Tables

**Table 1:** Benchmark prebiotic reaction networks for sugars, purines, and amino acids.

Reaction Network	Core Chemistry and Key Intermediates	Main Outputs and Notes
Formose reaction	Net: *n* CH_2_O -> sugars, C_n_H_2n_O_n_	Output: mixed sugars, C_2_ to C_8_+
	Key nodes: glycolaldehyde, glyceraldehyde	Limitation: ribose minor, unstable
	Steps: aldol addition, isomerization, tetrose to pentose	Bias: borate, silicates or clays, cycling
HCN oligomerization to purines	Net: n HCN -> oligomers	Output: adenine and other purines plus analogs
	Key nodes: AMN, DAMN	Limitation: insoluble HCN polymers
	Steps: cyclization, rearrangement to purines	Bias: UV, minerals, aqueous reactions
Miller type amino acids, Strecker synthesis	R-CHO + NH_3_ -> imine + H_2_O	Output: amino acids, glycine, alanine
	Imine + HCN -> aminonitrile Aminonitrile + 2 H_2_O -> alpha amino acid + NH_3_	Limitation: mix tracks aldehyde pool Bias: aqueous trap hydrolysis

Abbreviations: AMN, aminomalononitrile; DAMN, diaminomaleonitrile; HCN, hydrogen cyanide; UV, ultraviolet; R, organic substituent. Arrows indicate simplified network relationships, not balanced stoichiometry or yields.

## Data Availability

The original contributions presented in the study are included in the article, further inquiries can be directed to the corresponding author.

## Abbreviations

The following abbreviations are used in this manuscript:

ATP	adenosine triphosphate
CH_4_	methane
CO	carbon monoxide
CO_2_	carbon dioxide
DAMN	diaminomaleonitrile
DAP	diamidophosphate
DNA	deoxyribonucleic acid
Fe^2+^	ferrous ion
H_2_	molecular hydrogen
H_2_O	water
HCN	hydrogen cyanide
H_2_NCHO	formamide
Mg^2+^	magnesium ion
N_2_	molecular nitrogen
NH_3_	ammonia
PO_4_^3−^	orthophosphate
pH	negative logarithm of hydrogen ion activity, measure of acidity
RNA	ribonucleic acid
UV	ultraviolet
